# Pr^3+^ doping at the A-site of La_0.67_Ba_0.33_MnO_3_ nanocrystalline material: assessment of the relationship between structural and physical properties and Bean–Rodbell model simulation of disorder effects

**DOI:** 10.1039/c9ra03494c

**Published:** 2019-08-15

**Authors:** Ma. Oumezzine, Herbet Bezerra Sales, Ahmed Selmi, E. K. Hlil

**Affiliations:** Laboratory of Physical Chemistry of Materials, Faculty of Sciences of Monastir, University of Monastir 5019 Monastir Tunisia oumezzine@hotmail.co.uk; UEMA/CCT, Universidade Federal de Campina Grande – UFCG Campina Grande PB Brazil; Institut Néel, CNRS, Université J. Fourier B.P. 166 38042 Grenoble France

## Abstract

Bulk nanocrystalline samples of (La_1−*x*_Pr_*x*_)_0.67_Ba_0.33_MnO_3_ (0.075 ≤ *x* ≤ 0.30) manganites with a fixed carrier concentration are prepared by the sol–gel based Pechini method. Rietveld refinement of the X-ray diffraction patterns, shows the formation of single-phase compositions with rhombohedral symmetry. Upon Pr^3+^ doping at the A-site, the unit cell volume and the B–O–B bond angles are reduced. FTIR spectra present a prominent absorption peak of the in-phase stretching mode (B_2g_ mode) rising from the vibration of the Mn–O bond. Raman spectra at room temperature reveal a gradual shift toward lower frequencies in (E_g_) phonon mode with increasing Pr^3+^ concentration. The *M*(*T*) measurements shows a clear ferromagnetic (FM)–paramagnetic (PM) phase transition with increasing temperature. An increase in resistivity and activation energy and a decrease in the metal–semiconductor transition (*T*_M–SC_) and Curie temperatures (*T*_C_) was observed as a consequence of Pr^3+^ doping. The results are discussed according to the change of A-site-disorder effect caused by the systematic variations of the A-site average ionic radius 〈*r*_A_〉 and A-site-cation mismatch *σ*^2^, resulting in the narrowing of the bandwidth and the decrease of the mobility of e_g_ electrons. The magneto-transport behavior in the whole measured temperature and a magnetic field can be described by a percolation model, which is in agreement with the limited experimental data of the samples for *x* = 0.075, 0.15 and 0.30. The experimental results confirm that A-site substitution with Pr^3+^ destroys the Mn^3+^–O^2−^–Mn^4+^ bridges and weakens the double exchange (DE) interaction between the Mn^3+^ (t^3^_2g_e^1^_g_, *S* = 2) and Mn^4+^ (t^3^_2g_e^0^_g_, *S* = 3/2) ions. On the other hand, the Bean and Rodbell model has been successfully used to simulate the magnetization data of the samples with *x* = 0.15 and *x* = 0.22. The random replacement of La^3+^ by Pr^3+^ is shown to induce more disorder in the system, which is reflected in the increase of the fitted disorder parameter and spin value fluctuation. At a temperature close to room temperature, the maximum magnetic entropy change (Δ*S*_Max_) and the relative cooling power (RCP) of La_0.52_Pr_0.15_Ba_0.33_MnO_2.98_ are found to be, respectively, 1.34 J kg^−1^ K^−1^ and 71 J kg^−1^ for a 1.5 T field change.

## Abstract 2

Bulk nanocrystalline samples of (La_1−*x*_Pr_*x*_)_0.67_Ba_0.33_MnO_3_ (0.075 ≤ *x* ≤ 0.30) manganites with a fixed carrier concentration are prepared by the sol–gel based Pechini method.

## Graphical abstract

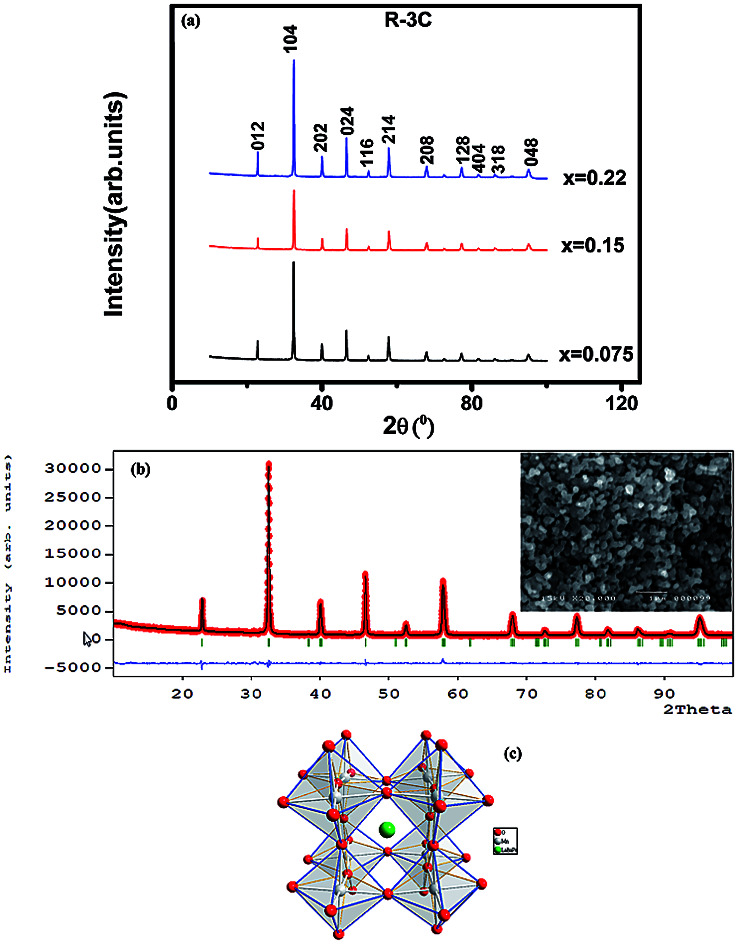

## 1 Introduction

The doped perovskite manganites with the general formula R_1−*x*_A_*x*_MnO_3_ (where R and A are trivalent rare earth and divalent alkaline earth ions, respectively) stimulate great scientific research because of their rich physical properties. In particular, these materials exhibit a remarkably rich variety of structural, magnetic, and transport properties because of couplings between spin and orbital moments.^1–4^ Doped lanthanum based manganites have been used in an enormous number of technological applications, including magnetic recording, high-density data storage, hard disks, magnetic sensors, spin-electronic devices, and magnetic refrigerants.^5–7^ These materials are of particular interest because of their chemical stability, the tunability of their Curie temperatures (*T*_C_) through doping, and their low synthesis cost. Barium-substituted lanthanum manganite (La_1−*x*_ Ba_*x*_MnO_3_) is among the existing colossal magnetoresistance (CMR) manganites displaying ferromagnetic behavior in a wide concentration range *x* with a maximum *T*_C_ well above room temperature for *x* = 0.33.^8^ Hence, the structure of La_1−*x*_ Ba_*x*_MnO_3_ can be derived from the cubic perovskite by tilting all oxygen octahedra about the [111] pseudo-cubic axes (a–a–a– tilt system). This system can be strongly modified by replacing a fraction *x* of the La^3+^ ions by the larger Ba^2+^ ions, resulting in a reduction of the Mn–O bond length (*d*_Mn–O_) and an increase of the Mn–O–Mn bond angle (*θ*_Mn–O–Mn_) towards 180°. Due to charge neutrality, replacing La by Ba causes the conversion of Mn^3+^ (t^3^_2g_e^1^_g_, *S* = 2) into Mn^4+^ (t^3^_2g_e^0^_g_, *S* = 3/2), which in turn introduces mobile e_g_ electrons in the manganite oxides. The mobile electrons are closely related to the ferromagnetic (FM) interactions between Mn^3+^ and Mn^4+^ (*i.e.*, the formation of Mn^3+^–O^2−^–Mn^4+^ networks) according to the double exchange (DE) interaction model,^9–11^ which is one of the mechanisms used to explain the magnetic and transport properties of these compounds. The significant changes in structure, magnetic and magnetoresistive (MR) properties of manganites can be achieved by varying particle size,^12–14^ oxygen stoichiometry^15^ and substituting cations at the A- or the B-sites.^4,16,17^

There are various methods to synthesize the manganites compounds involving solid state reaction, hydrothermal synthesis and Pechini sol–gel method. Pechini method has been used successfully to produce high-quality specimens due to these potential advantages such as better homogeneities, lower processing temperatures, short annealing times, high purity of materials and improved material properties.^18^ La_0.67_Ba_0.33_MnO_3_ (LBMO) has been one of the most appealing manganites, and in its bulk form, it has been found to exhibit by Mn-site substitution (B-site) in perovskite oxides, with other transition metal ions,^8,17,18^ a second order of magnetic phase transition. Besides, past studies on substituting praseodymium at the A-site of manganite are focused on polycrystalline ceramics.^19,20^

The objective of this work was to synthesize nanocrystalline samples of (La_1−*x*_Pr_*x*_)_0.67_Ba_0.33_MnO_3_ with an extended doping levels up to *x* = 0.30 and study the influence of praseodymium substitution at A-site on the crystal structural, magnetic, magneto-transport properties and magnetocaloric effect. Also, we aim to emphasize the interplay between experimental results and theoretical aspects of magnetization using Bean–Rodbell model and electrical resistivity adopting the percolation model.

## 2 Experimental details

### 2.1. Synthesis of samples

Nanocrystalline samples of (La_1−*x*_Pr_*x*_)_0.67_Ba_0.33_MnO_3_ (0.075 ≤ *x* ≤ 0.30) were synthesized using the Pechini sol–gel technique using highly pure metal nitrates as starting materials (>99.99% purity): La(NO_3_)_3_·6H_2_O, Pr(NO_3_)_3_·6H_2_O, Ba(NO_3_)_2_ and Mn(NO_3_)_2_·4H_2_O. The initial solution was prepared by mixing distilled water, nitrates (properly weighed according to the specific composition), citric acid (CA) (99.5% purity) and ethylene glycol (EG) (99.5% purity) in the following molar proportion 1 : 5 : 4 : 3. The resulting solution was heated by constant stirring at temperatures of 80 °C. After the evaporation of water at 80–100 °C, the viscosity of the solution increased and further heating led to the formation of polymeric resin. The resin was pre-calcined (673 K for 3 h) to eliminate the organic material, ground and calcined again (1073 K for 4 h) to eliminate the residual organic material. The obtained black powder was cold-pressed into pellets with a diameter of 13 mm and thickness of about 2–3 mm under a pressure of 5 tons per cm^2^. Subsequently, the powder was sintered at 1323 K for 12 hours in air.

### 2.2. Characterizations

#### 2.2.1. X-ray diffraction (XRD)

The samples were characterized using X-ray diffraction (XRD) to confirm the crystallinity, purity and single-phase formation of the samples of present investigation. The XRD patterns were further analyzed by employing Rietveld refinement technique (using Fullprof program), to estimate the lattice parameters, space groups, type of crystal system, Bragg reflections and other related statistics of the samples. Structural characterization using a ‘‘Panalytical X pert Pro’’ diffractometer with Cu K_α_ radiation (*k* = 1.5406 Å). Data for Rietveld refinement were collected in the range of 2*θ* from 10° to 120° with a step size of 0.017° and a counting time of 18 s per step.

#### 2.2.2. Iodometric titration

The Iodometric titration method was performed to estimate the Mn^4+^/Mn^3+^ ratio and oxygen stoichiometry of samples. Powders were weighed (about 100 mg) and dissolved in a mixture of 10 ml of 10 mass% potassium iodide aqueous solution and 2.5 ml of 2 M hydrochloric acid. Liberated iodine was titrated against standard sodium thiosulfate (0.04 N) solution using starch as an indicator.

#### 2.2.3. Surface morphology (FE-SEM)

The morphological properties of the samples were investigated by scanning electron microscopy (SEM) on a JSM-6400 apparatus working at 20 kV.

#### 2.2.4. DC electrical resistivity

Electrical resistivity measurements were carried out by standard four-probe method in the temperature range 5–300 K up to 5.0 T. The measurements were performed using the DC resistivity option in a Quantum Design physical property measurement system (PPMS).

#### 2.2.5. Magnetic measurement

The magnetization was measured in a field-cooled (FC) mode between 5 K and 400 K, under a magnetic field of 500 Oe, using a Quantum Design SQUID susceptometer, model MPMS-XL5. The isothermals *M versus H* at various temperatures around *T*_C_ have been measured in applied fields up to 5 T. These isothermals are corrected by a demagnetization factor *D* that has been determined by a standard procedure from low-field dc magnetization measurement at low temperatures (*H* = *H*_app_ − *DM*). The isothermal magnetization was performed after the sample was heated well above *T*_C_ for a long enough time, then cooled under zero field to the objective temperature.

## 3 Results

### 3.1. Structure and morphology studies

**Fig. 1:**
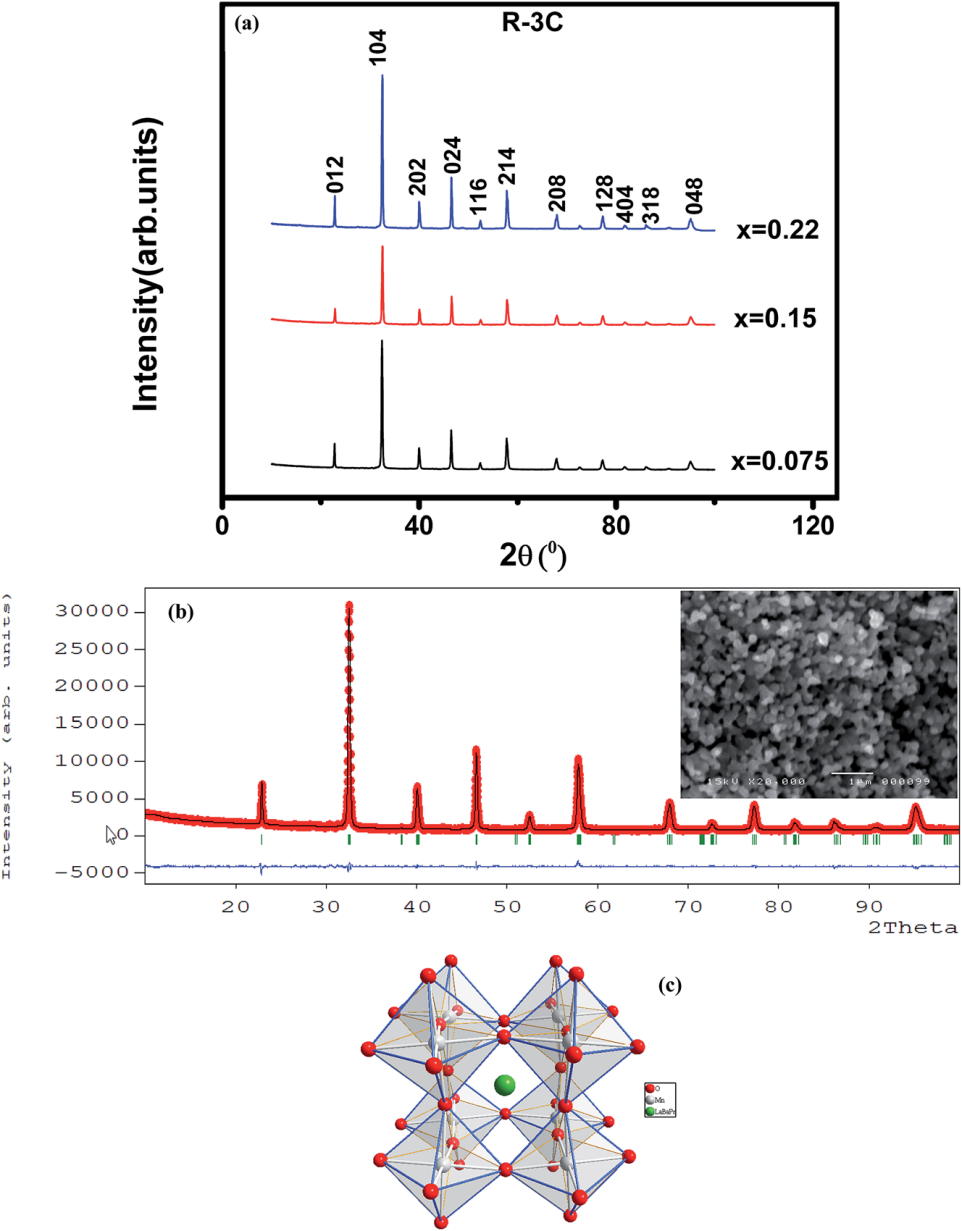
(a) XRD patterns of (La_1−*x*_Pr_*x*_)_0.67_Ba_0.33_MnO_3_ (*x* = 0.075, 0.15 and *x* = 0.22) compounds at room temperature. (b) Rietveld refinement profile of La_0.57_Pr_0.10_Ba_0.33_MnO_3_ performed using FULLPROF. Open circles correspond to experimental data and the lines are fits. Vertical bars represent the Bragg reflections for the space group *R*3̄*c*. The difference pattern between the observed data and fits is shown at the bottom. The right inset shows the FE-SEM image of *x* = 0.15 sample. (c) Crystal structure of La_0.57_Pr_0.10_Ba_0.33_MnO_3_ at room temperature in space group *R*3̄*c*.

The XRD patterns at room temperature for the (La_1−*x*_Pr_*x*_)_0.67_Ba_0.33_MnO_3_ (*x* = 0.075, *x* = 0.15 and *x* = 0.22) samples are shown in Fig. 1(a). It is evidence that all samples show typical reflections of the perovskite structure with rhombohedral symmetry and *R*3̄*c* (*Z* = 2) as a space group, no. 167. Hence, sharp peaks are clearly seen in all XRD patterns, indicating the studied samples to be highly crystalline. No traces of secondary phases were detectable, within the sensitivity limits of the experiment. The diffraction data were refined using the FullProf program by employing Rietveld powder diffraction technique.^21^ Background Rietveld refinements were fitted with a polynomial function; a pseudo-Voigt function was employed to model the peak shape. As a representative of the series, the refinement data of *x* = 0.15 composition is depicted in Fig. 1(b). The calculated results are shown in Table 1. Very good agreement between the calculated and the observed data is obtained. It may be seen that for all samples the residual factor is *R*_p_ ≤ 3.02, the weight residual factor is *R*_wp_ ≤ 3.71 and the goodness-of-fit factor is *χ*^2^ ≤ 2.85. These parameters confirm that the refinements are acceptable and the samples compositions are the same as their nominal compositions, including that the oxygen content was close to 3 for all the samples.^22^ The three equivalent positions (6a (0, 0, 1/4), 6b (0, 0, 0), and 18e (*x*, 0, 1/4)) in the rhombohedral unit cell are occupied by 6a (La^3+^, Pr^3+^, Ba^2+^), 6b (Mn^3+^, Mn^4+^), and 18e O^2−^ respectively. Fig. 1(c) shows crystal structure of La_0.57_Pr_0.10_Ba_0.33_MnO_3_ showing MnO_6_ octahedral generated with the help of program Diamond using refined cell parameters, space group and positional parameters of atoms. Detailed crystallographic parameters are listed in Table 1, where *a* and *c* are the hexagonal cell parameters, *V* is the unit cell volume, *B*_iso_ is the isotropic thermal parameter, *θ*_(Mn–O–Mn)_ is the bond angle, *d*_Mn−O_ is the bond length and *x* is the oxygen position. It is clearly noticeable that the average A-site radius and the cell parameters of the rhombohedral compounds are found to decrease with increase Pr doping concentration on the A-site as shown in Table 1. The average A-site ionic radius has been calculated using nine coordinated ionic radii given by Shannon.^23^ The observed behavior might be attributed to the fact that the substitution of a smaller Pr^3+^ ion (1.179 Å) at site La^3+^ ion (1.216 Å) compresses the unit cell in all the three directions, thus decreasing its volume. These lattice effects are similar to those observed in previous studies on the same A-site substitution with praseodymium.^19,24^ It should also be pointed out that a strong correlation exists between 〈*r*_A_〉 and the Goldschmidt's tolerance factor *t*_g_ defined as:1
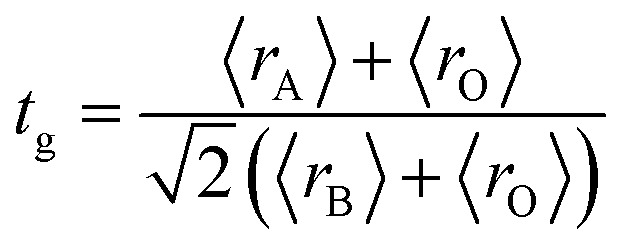
where 〈*r*_A_〉, 〈*r*_B_〉 and 〈*r*_O_〉 are respectively the average ionic radii of A and B perovskite sites and of the oxygen anions. When 〈*r*_A_〉 decreases, *t*_g_ also decreases and gives a lower symmetry arrangement by the tilting of the MnO_6_ octahedra. It is well-known that an orthorhombic structure is realized for *t*_g_ < 0.96, rhombohedral for 0.96 < *t*_g_ < 1, and cubic as *t*_g_ moves close to 1. In present study, it is found that *t*_g_ decreases from 0.9985 to nearly 0.9591 with increasing *x*, consistent with the experimental observation that the crystal structure of studied compounds is rhomobohedral. The decrease of *θ*_(Mn–O–Mn)_ bond angles (Table 1) increases the distortion of MnO_6_ octahedra that diminishes the strength of magnetic exchange interaction between the Mn^3+^ and Mn^4+^ ions. This could disfavor the long-range ferromagnetic order that results in shift of Curie temperature (*T*_C_) to lower temperature, which is comprehensively discussed in the next section. As a representation, the field emission scanning electron microscopy (FE-SEM) morphology for *x* = 0.22 is displayed in the inset of Fig. 1(b). The average grain size (GS)^25,26^ estimated is approximately 100 nm (±10 nm). From the iodometric titration method, the average ratio of Mn^3+^/Mn^4+^ is found to be fixed with an accuracy of ±0.03, while the oxygen content is close to stoichiometry (see Table 1).

**Table 1:** Detailed results of Rietveld refinement of (La_1−*x*_Pr_*x*_)_0.67_Ba_0.33_MnO_3_ (0 ≤ *x* ≤ 0.30) samples at room temperature

Sample	0 (ref. 18)	0.075	0.15	0.22	0.30
Structure type	Rhombohedral	Rhombohedral	Rhombohedral	Rhombohedral	Rhombohedral
Space group	*R*3̄*c*	*R*3̄*c*	*R*3̄*c*	*R*3̄*c*	*R*3̄*c*
**Lattice parameter**					
*a* (Å)	5.5304 (3)	5.5365 (3)	5.5371 (1)	5.5377 (1)	5.5385 (1)
*c* (Å)	13.5553 (3)	13.5034 (9)	13.4998 (5)	13.4965 (3)	13.4931 (3)
*V* (Å^3^)	359.06 (3)	358.47 (4)	358.45 (2)	358.44 (1)	358.42 (2)
*d*_Mn–O_ (Å)	1.9531 (1)	1.9594 (6)	1.9647 (7)	1.9658 (4)	1.9711 (1)
*θ*_(Mn–O–Mn)_(°)	176.50 (4)	172.06 (3)	168.42 (3)	167.77 (2)	164.11 (4)
*W*	0.0960	0.0950	0.0935	0.0933	0.0921
*t*_g_	0.985	0.982	0.979	0.976	0.972
〈*r*_A_〉 (Å)	1.396	1.387	1.378	1.370	1.356

**La/Ba**					
*x*	0.000	0.000	0.000	0.000	0.000
*y*	0.000	0.000	0.000	0.000	0.000
*z*	0.25	0.25	0.25	0.25	0.25
*B*_iso_ (Å^2^)	0.84 (0)	0.36 (1)	1.08 (2)	0.60 (1)	0.68 (2)

**Pr**					
*x*	—	0.000	0.000	0.000	0.000
*y*	—	0.000	0.000	0.000	0.000
*z*	—	0.000	0.000	0.000	0.000
*B*_iso_ (Å^2^)	—	0.168 (1)	0.889 (2)	0.410 (1)	0.517 (3)

**Mn**					
*x*	0.000	0.000	0.000	0.000	0.000
*y*	0.000	0.000	0.000	0.000	0.000
*z*	0.000	0.000	0.000	0.000	0.000
*B*_iso_ (Å^2^)	0.99 (3)	0.10 (2)	0.86 (3)	0.21 (2)	0.66 (3)

**O**					
*x*	0.480 (6)	0.524 (2)	0.535 (1)	0.462 (7)	0.495 (6)
*y*	0.000	0.000	0.000	0.000	0.000
*z*	0.25	0.25	0.25	0.25	0.25
*B*_iso_ (Å^2^)	2.60 (1)	1.76 (1)	1.39 (9)	0.50 (1)	0.63 (2)
*D*_W–H_ (nm)	—	75	95	80	93
Strain *ε* (%)	—	2.01 × 10^−3^	2.07 × 10^−3^	2.26 × 10^−3^	2.41 × 10^−3^
*σ* ^2^ (10^−2^ Å^2^)	0.267	0.547	0.726	0.878	1.058
Mn^4+^/Mn^3+^	0.468	0.463	0.472	0.466	—
Oxygen content	3.08	3.05	3.02	2.98	—

**Discrepancy factors (%)**					
*R*_wp_ (%)	8.71	3.53	3.71	4.03	3.91
*R*_p_ (%)	5.87	2.65	2.79	3.02	2.89
*R*_F_ (%)	4.85	0.60	1.41	1.20	1.07
*χ* ^2^ (%)	3.59	2.02	1.91	2.85	2.72

X-ray line profile analysis is a powerful and simple tool to identify the presence of dopant ion in the host lattice and quantify the microstructural parameters like size and lattice strain. Williamson–Hall (W–H) had proposed that the crystallite size and strain contributions to line broadening are independent to each other and it can be deduce in the following mathematical expression:^27^2
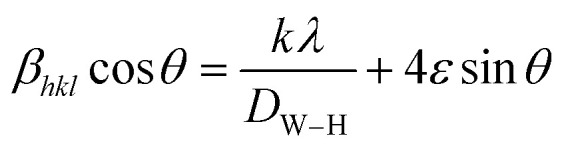
where *β*_*hkl*_ is the integral breadth (in radians). The *β* parameter was corrected for instrumental broadening. *λ* is the wavelength of the X-rays (Cu K_α_ radiation, *λ* = 1.5406 Å), *θ* is Bragg diffraction angle, *ε* is the lattice strain and *k* is the shape factor (*k* = 0.9). A plot is drawn by taking 4 sin *θ* along *X*-axis and *β*_*hkl*_ cos *θ* along *Y*-axis (not shown). The strain present in the material and the crystallite size are, respectively, extracted from the slope and the intercept of the linear fit made to the plot. The estimated values of the strain (*ε*) and the crystallite size (*D*_W–H_) are given in Table 1. It is clear from the table that the average crystallite size values are found to be in the range of 75–93 nm.

### 3.2. Fourier transform infrared spectroscopy (FTIR)

**Fig. 2:**
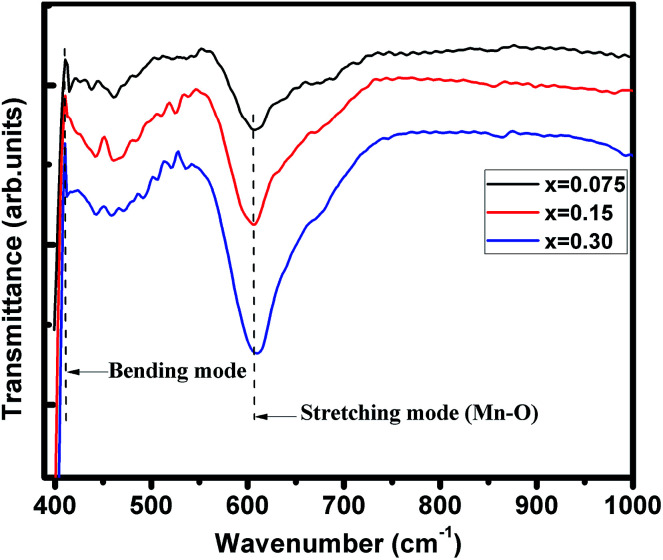
Infrared phonon spectra of (*x* = 0.075, 0.15 and 0.30) samples.

FTIR spectra of (La_1−*x*_Pr_*x*_)_0.67_Ba_0.33_MnO_3_ (where, *x* = 0.075, 0.15, and 0.30) samples recorded in the wavenumber range 400–1000 cm^−1^ are presented in Fig. 2. These metal oxygen bonds are subsequently organized into a MnO_6_ octahedral structure, as evidenced by the appearance of well-defined peaks. The higher frequency band at 610 cm^−1^ (for *x* = 0.30) corresponds to the in-phase stretching mode (B_2g_ mode) of Mn ion against the oxygen octahedron, which involves the internal motion of a change in Mn–O bond length.^28,29^ Since the increase in the concentration of Pr^3+^ ion with less ionic radii produces the shifting of wavenumbers (587 cm^−1^ for pristine sample^20^) to higher frequency, which is determined by a decrease of symmetry of the lattice. The increase in B–O vibration frequency for ABO_3_ structure indicates a strong coupling constant and hence the shorter bond lengths/decrease in lattice volume, supporting the XRD results discussed above. The band at 410 cm^−1^ corresponds to the *E*_g_-symmetry mode associated to an internal bending mode of the MnO_6_ octahedra. These two bands are related to the environment surrounding the MnO_6_ octahedra in the ABO_3_ perovskite and confirms the formation of perovskite structure,^30,31^ which is in agreement with the XRD results.

### 3.3. Raman spectroscopy

Raman spectroscopy is a powerful and sensitive tool for understanding crystal symmetry, the local structural distortion and its dependence on doping. Our samples crystallize in rhombohedral structure, space group *R*3̄*c* (D^6^_3d_), *Z* = 2. This structure can be obtained from the simple cubic perovskite by the rotation of the adjacent MnO_6_ octahedra in the opposite directions around the [111] cubic direction. According to the group theory, for this structure, thirty vibrational degrees of freedom at the *Γ* point are distributed among the irreducible representation as:

3 (*Γ*(D^6^_3d_) = 2A_1u_ + 3A_2g_ + A_1g_ + 4A_2u_ + 4E_g_ + 6E_u_)

The rhombohedral distortion gives rise to five Raman active modes.

**Fig. 3:**
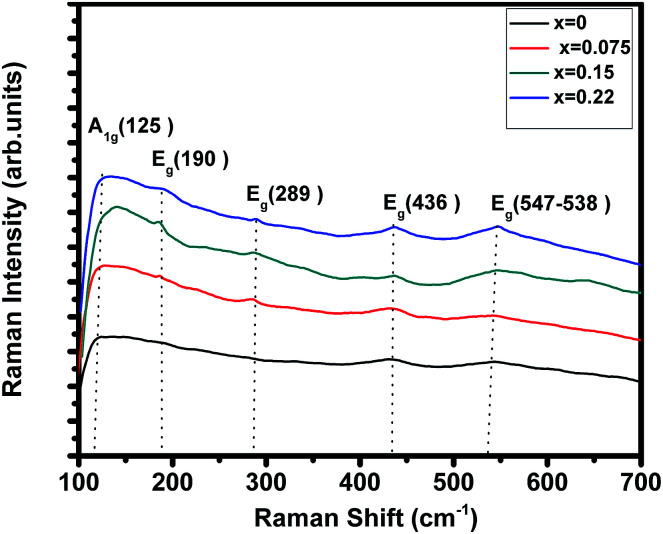
Raman spectrum of (La_1−*x*_Pr_*x*_)_0.67_Ba_0.33_MnO_3_ (*x* = 0, 0.075, 0.15 and *x* = 0.22) samples.

Room temperature Raman spectrum of (La_1−*x*_Pr_*x*_)_0.67_Ba_0.33_MnO_3_ (where, *x* = 0, 0.075, 0.15, and 0.22) samples in the frequency range of 100–700 cm^−1^ is shown in Fig. 3. Five vibration modes have been identified, one (A_1g_) and four (E_g_). These broad bands are located at 125 (A_1g_), 190 (E_g_), 289 (E_g_), 436 (E_g_) and 547 (E_g_) cm^−1^, which are associated with rotational-, bending-, and stretching-like vibrations of the MnO_6_ octahedra, respectively.^32,33^ In this work, we underline the (E_g_) mode (approx. 547 cm^−1^) allowed for the symmetric stretching vibration of oxygen in MnO_6_ octahedra. This mode shows a substantial shift toward lower frequencies (a downshift of about 10 cm^−1^) as a function of Pr concentration. These shifts are related to the change in the average Mn–O distance.^34^

### 3.4. Magnetic properties

**Fig. 4:**
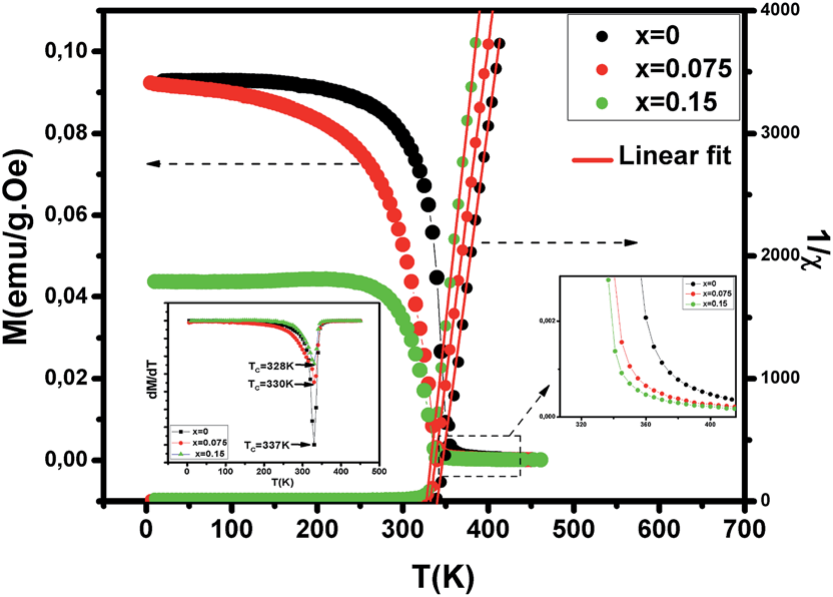
Temperature dependence of the magnetization for (La_1−*x*_Pr_*x*_)_0.67_Ba_0.33_MnO_3_ (*x* = 0, 0.075 and *x* = 0.15) measured in field cooling (FC) mode at an applied magnetic field of *μ*_0_*H* = 500 Oe and temperature dependence of the inverse of magnetic susceptibility 1/*χ* for selected samples with *x* = 0, 0.075 and *x* = 0.15. The red line presents the linear fit at high temperature. Left inset shows the temperature derivative d*M*/d*T*. Right inset: zoomed-in view of magnetization above *T*_C_.

To investigate the magnetic properties of (La_1−*x*_Pr_*x*_)_0.67_Ba_0.33_MnO_3_ (0 ≤ *x* ≤ 0.30) nanocrystalline, we performed temperature dependent field cooled magnetization measurements (*M*–*T*) from 400 K to 5 K at 500 Oe magnetic field (Fig. 4). A transition from a low-temperature ferromagnetic phase to a high temperature paramagnetic phase is evident. The Curie temperature *T*_C_ is the temperature at which the absolute value of d*M*/d*T* is maximum (see the left inset of Fig. 4), are summarized in Table 2. The ∼337 K transition for the pristine compound is shifted toward room temperature with increasing Pr concentration, until in the *x* = 0.30 composition occurs at *T*_C_ = 309 K. The effective e_g_ bandwidth *W*,^35^ determined by the overlapping of Mn 3d and O 2p orbitals, strongly depends on ionic radii and the structural distortion. In this work, both Pr^3+^ and La^3+^ are trivalent positive ions, the substitution of La^3+^ by Pr^3+^ does not change the charge carrier density. As we know, the smaller average ionic radius 〈*r*_A_〉 decreases the Mn–O–Mn bond angles and increases the Mn–O bond lengths,^36,37^ which weakens the hopping integral of e_g_ electrons and attenuates the DE interaction. The initial decrease of *T*_C_ (see Table 2) is related to the reduce of the bandwidth. On the one hand, we can see in Fig. 4 that the magnitude of magnetization in the ferromagnetic region is decreased (*i.e.*, when Pr^3+^ content increases, as discussed above), which is consistent with results reported earlier.^38–40^ Moreover, other studies of A-site doped manganites^41,42^ also reveal that the mismatch in the size of the A-cation (*σ*^2^) influences the *T*_C_. Thus, we have to consider *σ*^2^, which is defined by the relations:

4 *σ*^2^ = ∑*x*_*i*_*r*^2^_*i*_ − 〈*r*_A_〉^2^

where *x*_*i*_
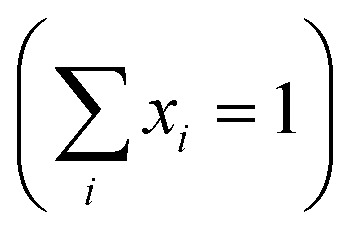
 is the fractional occupancy and *r*_A_ is calculated as: *r*_A_ = 0.67 × (1 − *x*) × *r*_La^3+^_ + 0.67 × *x* × *r*_Pr^3+^_ + 0.33 × *r*_Ba^2+^_. The substitution of smaller Pr^3+^ for larger La^3+^ cations causes a decrease in the average A-site cationic radius, while *σ*^2^ increases (the intrinsic size disorder) (see Table 1). This enhancement in the mismatch in the crystal structure induces a lattice strain by causing a random displacement of oxygen atoms from their average crystallographic positions, thereby resulting in a distortion of the MnO_6_ octahedra, and hence the e_g_ electrons are localized. So, these changes lead to decrease in *T*_C_ value of the compounds by weakening double-exchange interaction. To get a clear knowledge about the magnetic interaction for (La_1−*x*_Pr_*x*0.67_Ba_0.33_MnO_3_ series, the inverse susceptibility (1/*χ*) *versus* temperature (*T*) curves are plotted as shown in Fig. 4. A typical Curie–Weiss behaviour is observed above the *T*_C_ where 1/*χ* is changing almost linearly with the temperature which can be fitted by 
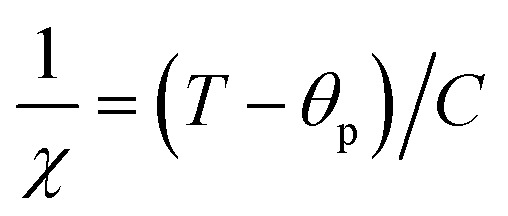
, where *C* is the Curie constant and *θ*_p_ is the paramagnetic Curie–Weiss temperature. By fitting the linear region, the Curie–Weiss temperatures *θ*_p_, which are an indication of the nature and strength of coupling in the structure, and *C* were obtained. It is evident that *θ*_p_ is always positive for all three samples, indicating the existence of FM exchange interaction between spins. Next, the experimental effective paramagnetic moments *μ*^exp^_eff_, are derived for each sample using the following equation:5
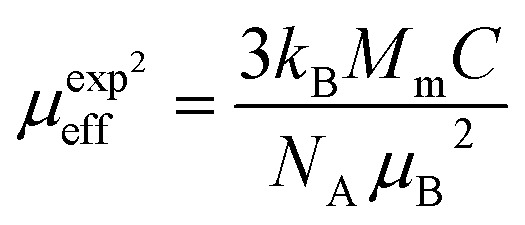
where *k*_B_ = 1.38016 × 10^−23^ J K^−1^ is the Boltzmann constant, *N*_A_ = 6.023 × 10^23^ mol^−1^ is Avogadro's number, *M*_m_ is the molecular weight and *μ*_B_ = 9.274 × 10^−21^ emu is the Bohr magnetron. These values, together with the Curie–Weiss temperature, are listed in Table 2. The theoretical effective paramagnetic moment is calculated based on the chemical formula of (La_1−*x*_Pr_*x*_)^3+^_0.67_Ba^2+^_0.33_Mn^3+^_0.67_Mn^4+^_0.33_O^2−^_3_, using the following expression:6
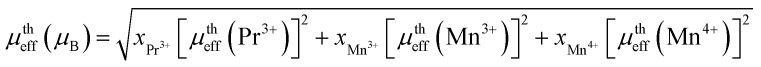


**Table 2:** Values of the Curie temperature *T*_C_, the Curie constant *C*, the Curie–Weiss temperature *θ*_CW_ and the experimental and theoretical effective paramagnetic moment (*μ*^exp^_eff_) and (*μ*^th^_eff_) for (La_1−*x*_Pr_*x*_)_0.67_Ba_0.33_MnO_3_ (0 ≤ *x* ≤ 0.30)

(La_1−*x*_Pr_*x*_)_0.67_Ba_0.33_MnO_3_	*T*_c_ (K)	*θ*_CW_ (K)	*C* (emu mol^−1^ Oe^−1^ K)	*μ* ^th^_eff_ (*μ*_B_)	*μ* ^exp^_eff_ (*μ*_B_)
0.00	337	340	3.60	4.58	6.03
0.075	330	333	3.96	4.64	5.63
0.15	328	330	3.30	4.72	5.14
0.22	319	323	4.42	4.78	5.95
0.30	309	308	5.64	4.85	6.72

The spin-only magnetic moments for free Mn^3+^, Mn^4+^ and Pr^3+^ are 4.89*μ*_B_, 3.87*μ*_B_, 3.58*μ*_B_, respectively. Thus, both the experimental *μ*^exp^_eff_ and theoretical *μ*^th^_eff_ values of the effective moment are given in Table 2. As it can be seen from the Table 2, the experimental (*μ*^exp^_eff_) values are in the range of ∼6.03–6.72*μ*_B_, and thus are little larger than the theoretical values. Such a difference in (*μ*_eff_) values may be ascribed to the appearance of short-range FM interactions in the paramagnetic state (above *T*_C_), which is commonly observed in manganites.^18,20,43^

### 3.5. The Bean–Rodbell model

In order to study the nature of the magnetic transition, we have applied the Bean–Rodbell model to our magnetization data for (La_1−*x*_Pr_*x*_)_0.67_Ba_0.33_MnO_3_ (*x* = 0.15, and 0.22). Manganite materials^18,44^ with second- and first-order phase transition have been adequately interpreted using this model, which describes in particular the magnetovolume interactions.^45^ The model considers the dependence of the exchange interaction on the interatomic distance. This dependence is phenomenologically described by considering the dependence of the critical magnetic phase-transition temperature on the volume change in the following way:7
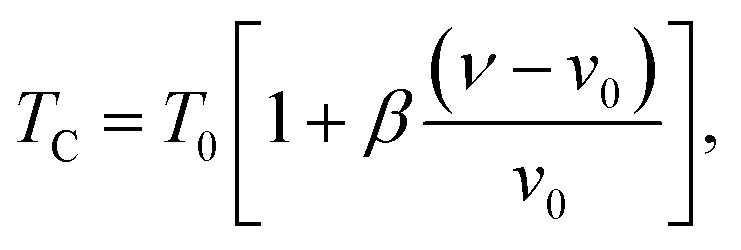
where *ν* is the volume and *ν*_0_ is the equilibrium volume obtained in the absence of magnetic interaction. *T*_0_ is the magnetic ordering temperature in the absence of deformations. The parameter *β* represents the slope of the dependence of the Curie temperature (*T*_C_) on the cell deformation.

**Fig. 5:**
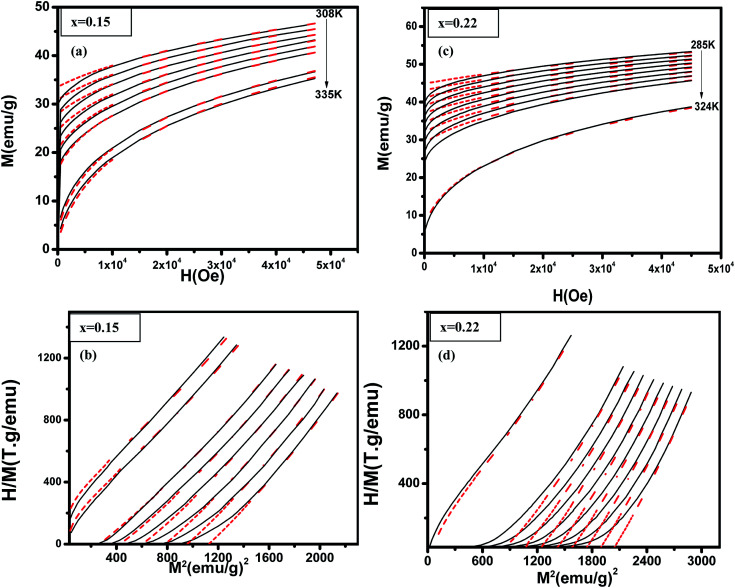
(La_1−*x*_Pr_*x*_)_0.67_Ba_0.33_MnO_3_ (*x* = 0.10 and *x* = 0.15) experimental (black lines) and simulated (red dots) curves showing ((a) and (c)) magnetization as a function of applied magnetic field, at the indicated temperature values and ((b) and (d)) corresponding *H*/*M vs. M*^2^ Arrott plots.

Considering a material with compressibility *K*, spin *J* and spin density *N*, one defines the *η* parameter:8
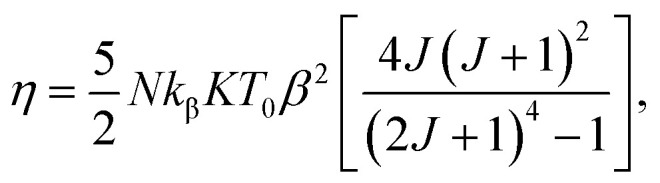
where *k*_β_ is the Boltzmann constant. Bean and Rodbell proved that in this model this parameter governs the nature of the magnetic phase transition. In the absence of external pressure, for 0 ≤ *η* < 1 the transition is second order type while for *η* > 1 the transition is purely first order type,^45^ with coupled volume and magnetization discontinuities at specific field and temperature values. Note that, the model is a modified form of the Bean–Rodbell model extended to include spin clustering *via* the parameter *J*. The experimental data can only be well described if a Gaussian distribution of values with variable full width at half maximum (FWHM), accounting for sample inhomogeneity, is incorporated into the model. The parameters *η*, *J*, *T*_0_, and its FWMH, are tuned in order to provide a best fit to experimental curves such as *M vs. H*, *M vs. T*, and, *H*/*M vs. M*^2^.^18^Fig. 5 shows examples data for (La_1−*x*_Pr_*x*_)_0.67_Ba_0.33_MnO_3_ (*x* = 0.15, and 0.22) and best fit curves. We see a good match especially at high field and high magnetization between measurements and simulated data. As the model assumes a homogeneous and isotropic system, effects such as magnetic domains, anisotropy, and demagnetization are not taken into account, justifying the higher deviation between experimental data and simulations at lower fields.^46^Table 3 shows the parameters obtained from these simulations. The second-order transition of our samples is confirmed by their *η* parameter value (*η* < 1).^45^ The partial substitution of La^3+^ by magnetic ions Pr^3+^ does not alter the ratio of Mn^3+^/Mn^4+^ ions but results in a more distorted structure. These changes lead to suppression of the ferromagnetism which affect the long-range ferromagnetic order. To our knowledge, the relationship between the Pr^3+^ doping at A-site of lanthanum manganite and disorder effects has not been reported before in manganites. From Table 3, it can be observed that Pr^3+^ doping induces more disorder in the system, as it can be seen from the evolution of the disorder parameter and spin value fluctuation.

**Table 3:** Parameters extracted from Bean–Rodbell based analysis for La_0.60_Pr_0.07_Ba_0.33_MnO_3.02_ and La_0.52_Pr_0.15_Ba_0.33_MnO_2.98_

Composition	La_0.60_Pr_0.07_Ba_0.33_MnO_3.02_	La_0.52_Pr_0.15_Ba_0.33_MnO_2.98_
**Mean-field and Bean–Rodbell analysis**		
*M*_Sat_	88.80	88.57
*T*_c_ (K)	329	320
*η*	0.38	0.41
Magnetic spin clustering (no. ions)	4.90	5.70
*T*_c_ FWHM disorder (K)	10	22

### 3.6. Prediction of the magneto-transport properties using percolation model

**Fig. 6:**
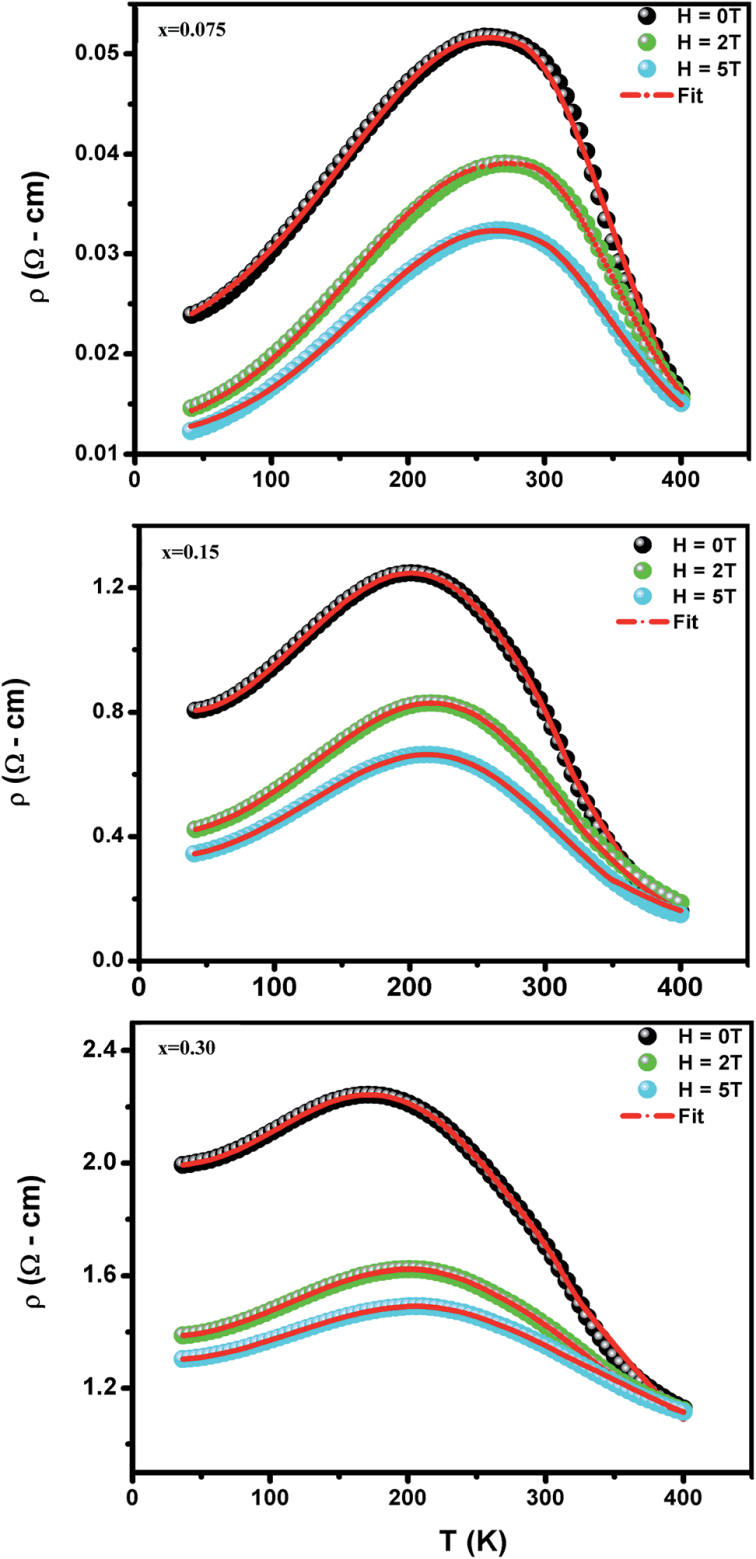
Temperature dependence of the resistivity *ρ* (*T*,*H*) at without and with different applied magnetic fields for (La_1−*x*_Pr_*x*_)_0.67_Ba_0.33_MnO_3_ with *x* = 0.075, 0.15 and *x* = 0.30. Red solid line corresponds to fit by eqn (9).

**Fig. 7:**
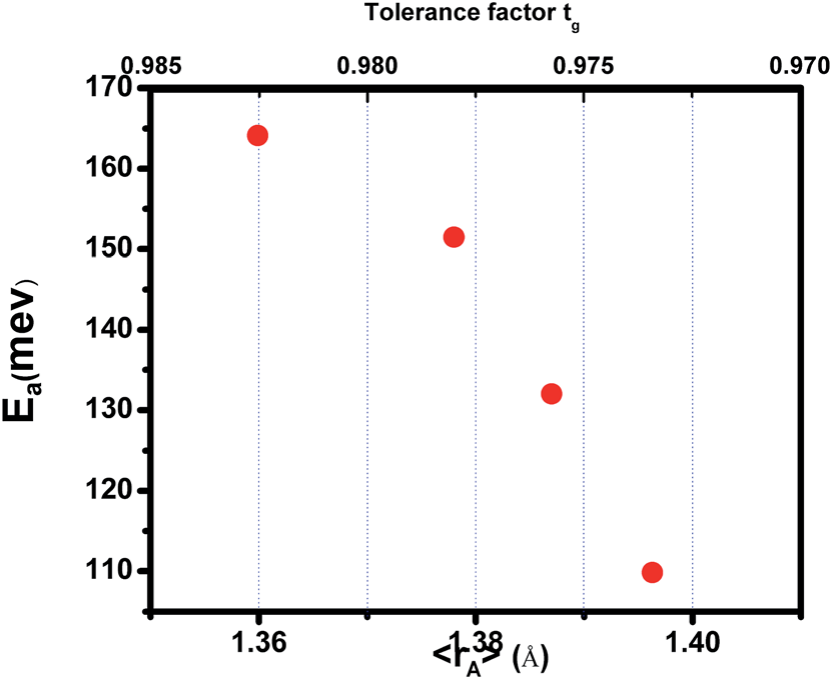
Activation energy *vs.* the tolerance factor *t*_g_ and the average ionic radius of the A-site 〈*r*_A_〉 for (La_1−*x*_Pr_*x*_)_0.67_Ba_0.33_MnO_3_ (0 ≤ *x* ≤ 0.30).

To investigate the effect of substitution with magnetic ions (*i.e.*, case of Pr^3+^) at A-site on the electronic transport properties of the samples, the temperature dependence of electrical resistivity measured both in presence and in absence of magnetic field (up to 5.0 T) on (La_1−*x*_Pr_*x*_)_0.67_Ba_0.33_MnO_3_ (*x* = 0.075, 0.15 and 0.30) samples are shown in Fig. 6(a–c). All the studied samples exhibit metallic behavior below the metal–semiconductor transition temperature (*T*_M–SC_) and semiconductor-like features above *T*_M–SC_. For the pristine sample, the metal–semiconductor transition is observed at the temperature (*T*_M–SC_) of about 340 K.^18^ The values of *T*_M–SC_ are found to decrease by 50% with increasing Pr^3+^ concentration (with *x* up to *x* = 0.30), supporting the XRD and magnetic results discussed above. Resistivity in the entire temperature range increases with increase in Pr^3+^ concentration, which can be attributed to the weakening of the DE interaction between the Mn^3+^ (t^3^_2g_e^1^_g_, *S* = 2) and Mn^4+^ (t^3^_2g_e^0^_g_, *S* = 3/2) *via* the intervening oxygen. On the other hand, the resistivity at a given temperature is found to decrease with increasing field and that *T*_M–SC_ values (see Table 4) are found to move towards high temperature side with increasing magnetic field. Praseodymium substitution at A-site may favor the charge carrier delocalization induced by the magnetic field, which suppresses the resistivity and consequently leads to the local ordering of the electron spins. Due to this ordering, the ferromagnetic metallic (FM-M) state may suppress the paramagnetic semiconducting (P-SC) regime resulting in complete polarization of conduction electrons (e^1^_g_) inside the magnetic domains and, thus are easily transferred between the pairs Mn^3+^ and Mn^4+^*via* oxygen. To elucidate the carrier transport behavior in the whole measured temperature and a magnetic field, we attempted to fit the magneto-resistance of (La_1−*x*_Pr_*x*_)_0.67_Ba_0.33_MnO_3_ (*x* = 0.075, 0.15 and 0.30) samples according to the percolation model.^47^ This model assumes the materials to be composed of ferromagnetic and paramagnetic regions and semiconductor-like transport properties are exhibited in the paramagnetic region, whereas metallic transport always show up in the ferromagnetic region. The relationship applied in the prediction of the magneto-transport can be expressed as follows9
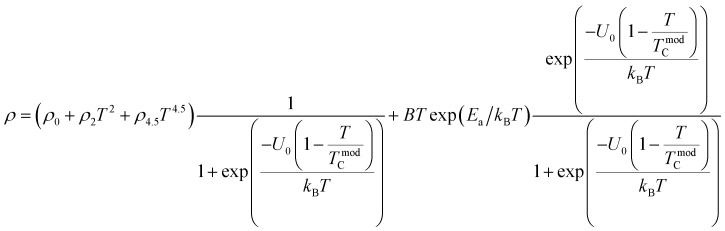
where *T*^mod^_C_ (adjustable parameter) means a temperature in the vicinity of what the resistivity has a maximum value.^47^ All other parameters, *viz.*, *ρ*_0_, *ρ*_2_, *ρ*_4.5_, and *E*_a_ are kept fixed to their respective values obtained independently for the metallic-ferromagnetic (*T* < *T*_M–SC_) and semiconductor-paramagnetic (*T* > *T*_M–SC_) regions (see Table 4). The experimental data in Fig. 6 were fitted using eqn (9). Fitting lines are shown in Fig. 6 and the results are presented in Table 4. The results calculated from eqn (9) agree well with the experimental data. However, the activation energy (*E*_a_) in the absence of an external magnetic field of the samples is extracted in the semiconductor-like conducting temperature region (well above *T*_M–SC_) in terms of a magnetic polaron picture^48^ (see Fig. 7). It was further observed that *E*_a_ in the transport process of the carriers increases with decreasing 〈*r*_A_〉 and/or *t*_g_, implying the decrease of the localization length and the reduction of the carrier mobility, which is intimately related to the localization of carriers and the destruction of DE interaction arising from Pr-doping at A-site. Thus, are in accordance with the structural and magnetic properties discussed in the previous sections.

**Table 4:** Best fit parameters of the electrical resistivity *ρ* (*T*,*H*) for (La_1−*x*_Pr_*x*_)_0.67_Ba_0.33_MnO_3_ (*x* = 0.075, 0.15 and *x* = 0.30) using (eqn (9)). Metal–semiconductor transition temperature *T*_M–SC_, and correlation factors *R*^2^

Sample	*x* = 0.075			*x* = 0.15			*x* = 0.30		
	0 T	2 T	5 T	0 T	2 T	5 T	0 T	2 T	5 T
*ρ*_0_ (Ω cm)	0.017	0.014	0.011	0.788	0.544	0.362	2.32 1.62	1.48	
*ρ*_2_ (Ω cm K^−2^)	5.44 × 10^−6^	5.22 × 10^−6^	5.08 × 10^−6^	8.72 × 10^−5^	5.96 × 10^−5^	3.53 × 10^−5^	5.43 × 10^−4^	3.88 × 10^−4^	1.65 × 10^−4^
*ρ*_4.5_ (Ω cm K^−4.5^)	2.95 × 10^−13^	2.35 × 10^−13^	1.74 × 10^−13^	7.76 × 10^−12^	4.66 × 10^−12^	2.94 × 10^−12^	6.19 × 10^−12^	2.43 × 10^−12^	1.63 × 10^−12^
*T*_M–SC_ (K)	256	266	270	201	211	215	171	201	206
*R* ^2^ (%)	0.998	0.995	0.997	0.972	0.961	0.988	0.998	0.968	0.988

### 3.7. Magnetocaloric effect of La_0.52_Pr_0.15_Ba_0.33_MnO_2.98_

**Fig. 8:**
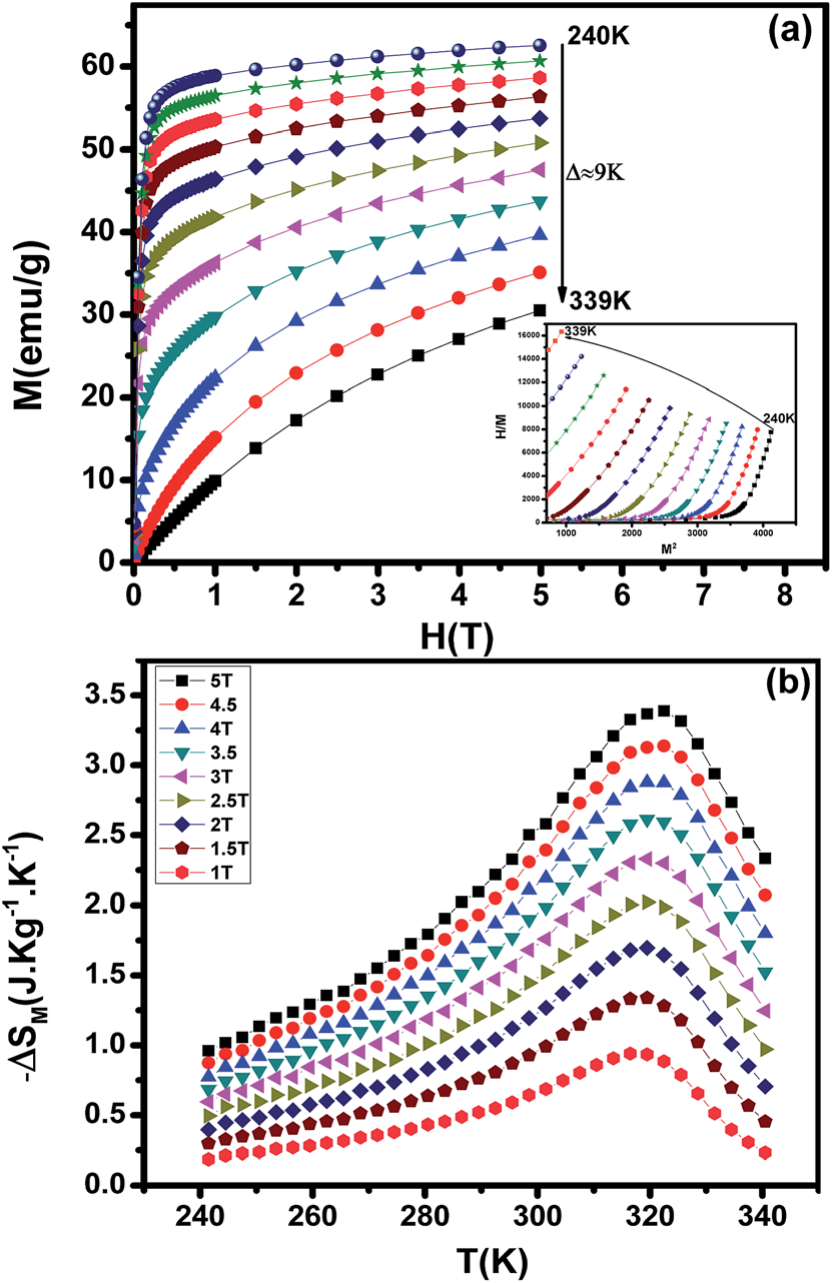
(a) Isothermal magnetization *versus* magnetic field around *T*_C_ of La_0.52_Pr_0.15_Ba_0.33_MnO_2.98_. Inset shows Arrott plot of *μ*_0_*H*/*M vs. M*^2^ at different temperatures. (b) The temperature dependence of the magnetic entropy change (Δ*S*_M_) under different applied magnetic fields.

MCE is an intrinsic property of magnetic materials. It is the response of the material toward the application or removal of a magnetic field. This response is maximized when the material is near its magnetic ordering temperature. The magnetization *M* as a function of the applied magnetic field, at various temperatures, is shown in Fig. 8(a). At the lowest temperatures, the magnetization saturates rapidly due to an easy orientation of the spins under the action of the applied field. No magnetic hysteresis is found around the transition temperature, suggesting that the material is a soft ferromagnetic. To assess the nature of magnetic phase transitions, Arrott plots^49^ (*μ*_0_*H*/*M versus M*^2^) were constructed based on the *M*–*H* data (Inset of Fig. 8(a)). All of the *M*^2^*vs. μ*_0_*H*/*M* curves show positive slopes without inflexion points, which is characteristic of second order transitions according to the Banerjee criterion.^50^ This feature is in agreement with Bean–Rodbell model analysis. The magnetic entropy changes, Δ*S*_M_, of La_0.52_Pr_0.15_Ba_0.33_MnO_2.98_ has been calculated using the Maxwell relation^51^ and is plotted in Fig. 8(b) as a function of temperature and field. The maximum value of magnetic entropy change Δ*S*_Max_ is found to be around *T*_C_ and it increases with increasing the magnetic applied field due to the enhancement of FM interactions.

At a Δ*S*_M_ of 5 T, the maximum value of the magnetic entropy change Δ*S*_M_ is found to be about 3.39 J kg^−1^ K^−1^ (1.34 J kg^−1^ K^−1^ for 1.5 T) and the estimated relative cooling power (RCP), which is considered as the efficiency of magnetocaloric materials based on the magnetic entropy change, is found to be 251 J kg^−1^ (71 J kg^−1^ for 1.5 T). The RCP value is calculated from the product of the peak entropy change times the full width at half maximum. These values are about 61% of those of pure Gd, the prototype magnetic refrigerant material (RCP = 410 J kg^−1^).^52^ For comparison, the obtained values are higher than the observed in manganite polycrystalline, La_0.40_ Pr_0.30_Sr_0.30_MnO_3_ (ref. 53) and La_0.55_ Pr_0.15_Sr_0.30_MnO_3_.^53^ From these results, we can estimate that our material is potential candidates to magnetic refrigeration applications around room temperature.

## 4 Conclusion

The influence of praseodymium substituting at La-site in (La_1−*x*_Pr_*x*_)_0.67_Ba_0.33_MnO_3_ (0.075 ≤ *x* ≤ 0.30) has been investigated, in structural, magnetic and electrical transport properties. The samples were synthesized using the Pechini sol–gel method. Rietveld refinement of XRD patterns shows that all samples crystallized in a rhombohedral structure with *R*3̄*c* space group. FTIR and Raman measurements confirms the perovskite structure of all the samples. The substitution of Pr^3+^ changes A-site average cationic radius and decreases the lattice parameters, Curie temperatures (*T*_C_), metal–semiconductor transition (*T*_M–SC_), and the magnitude of magnetization in the ferromagnetic region. It is observed that doping of Pr induces an increase in polaron activation energy. This fact indicates that Pr doping enhances electronic localization. Numerical simulations, in the framework of the molecular mean field model incorporating the Bean–Rodbell magnetovolume coupling were performed. We have found that Pr^3+^ doping on A-site leads to more chemical/structural disorder in second-order magnetic system. On the other hand, the behavior of *ρ* (*T*,*H*) of these samples in a wide range of temperatures and magnetic fields can be explained using the phenomenological model based on the phase segregation mechanism (percolation model).

Around room temperature, the La_0.52_Pr_0.15_Ba_0.33_MnO_2.98_ sample exhibit a sizable magnetic entropy change of 1.34 J kg^−1^ K^−1^ and a RCP of 71 J kg^−1^ under a magnetic field change of 1.5 T, making this compound a suitable candidate for active magnetic refrigeration.

## Conflicts of interest

There are no conflicts to declare.

## Supplementary Material
